# RAM score is an effective predictor for early mortality and recurrence after hepatectomy for hepatocellular carcinoma

**DOI:** 10.1186/s12885-017-3748-9

**Published:** 2017-11-09

**Authors:** Heng-Yuan Hsu, Ming-Chin Yu, Chao-Wei Lee, Hsin-I Tsai, Chang-Mu Sung, Chun-Wei Chen, Shu-Wei Huang, Cheng-Yu Lin, Wen-Juei Jeng, Wei-Chen Lee, Miin-Fu Chen

**Affiliations:** 1 Department of Surgery, Chang Gung Memorial Hospital, No. 5, Fuxing St., Guishan Dist., Taoyuan, 333 Taiwan, Republic of China; 2 College of Medicine, Chang Gung University, Taoyuan, Taiwan, Republic of China; 3 Graduate Institute of Clinical Medical Sciences, Chang Gung University, Taoyuan, Taiwan, Republic of China; 4 Department of Anesthesiology, Chang Gung Memorial Hospital, No. 5, Fuxing St., Guishan Dist., Taoyuan, 333 Taiwan, Republic of China; 5 Department of Gastroenterology and Hepatology, Chang Gung Memorial Hospital, No. 5, Fuxing St., Guishan Dist., Taoyuan, 333 Taiwan, Republic of China

**Keywords:** Early mortality, Early recurrence, Short term outcome, Hepatectomy, Liver resection, Hepatocellular carcinoma, Hepatoma, RAM score

## Abstract

**Background:**

Liver resection had been regarded as a standard treatment for primary hepatocellular carcinoma (HCC). However, early mortality and recurrence after surgery were still of major concern. RAM (Risk Assessment for early Mortality) scoring system is a newly developed tool for assessing early mortality after hepatectomy for HCC. In this study, we compared RAM scoring system with ALBI and MELD scores for their capability of predicting short-term outcome.

**Methods:**

We retrospectively reviewed patients with hepatocellular carcinoma who were treated with hepatectomy at Chang Gung Memorial Hospital between 1986 and 2015. Their clinical characteristics and perioperative variables were collected. We applied RAM, albumin-bilirubin (ALBI), and model for end-stage liver disease (MELD) scoring systems to predict early mortality and early recurrence in HCC patients after surgery. We investigated the discriminative power of each scoring system by receiver operating characteristic (ROC) curve and area under the ROC curve (AUC).

**Results:**

A total of 1935 patients (78% male) who underwent liver resection for HCC were included in this study. The median follow-up period was 41.9 months. One hundred and forty-nine patients (7.7%) died within 6 months after hepatectomy (early mortality). All the three scoring systems were effective predictor for early mortality, with higher score indicating higher risk of early mortality (AUC of RAM = 0.723, *p* < 0.001; AUC of ALBI = 0.682, *p* < 0.001; AUC of MELD = 0.590, *p* = 0.002). Cox regression multivariate analysis demonstrated that the RAM class was the most significant independent predictor of early mortality after surgery, while MELD grade failed to discriminatively predict early mortality. In addition to early mortality, the RAM score was also predictive of early recurrence in HCC after surgery.

**Conclusions:**

This study demonstrated that RAM score is an effective and user-friendly bedside scoring system to predict early mortality and early recurrence after hepatectomy for HCC. In addition, the predictive capability of RAM score is superior to ALBI and MELD scores. Further study is warranted to validate our findings.

## Background

Hepatocellular carcinoma (HCC) is the most common primary malignancy of the liver and the second most common reason of cancer-associated death worldwide [1]. In Taiwan, it is the second most common cause of cancer death and causes more than 8000 deaths each year [2]. Surgical resection remains the most effective therapy in selected patients, but the coexisting underlying liver diseases, such as chronic hepatitis B or C and alcoholic liver disease, had limited the extent and feasibility of liver resection. Earlier before 1980s, liver resections in the presence of liver cirrhosis was associated with a relatively high mortality rate in the range of 10–30%, and were therefore largely limited to minor resections [3–10]. With improvements in patient selection, surgical techniques and postoperative care, the mortality rate has improved dramatically in recent decades [3, 11–13]. Recent literature has reported a 30-day surgical mortality rate of 1.8% and in-hospital surgical mortality rate of 2.9% after liver resection for HCC [14]. Despite the tremendous decline in immediate postoperative mortality rate, the same study strikingly reported that many more patients (11.3%) would eventually expire by the 6th postoperative month (early mortality). Notably, surgeons still face great challenges for patients to survive long enough for scheduled follow-up visit or adjuvant therapy after such major operations. To optimize patients’ outcome, it is therefore of paramount significance to predict the occurrence of early mortality after liver resection for HCC.

Model for end-stage liver disease (MELD) score was initially designed to predict survival in patients undergoing transjugular intrahepatic portosystemic shunts (TIPS) for either prevention of variceal rebleeding or for treatment of refractory ascites [15]. In addition to predicting survival after TIPS, MELD score has also been regarded as significant prognostic indicators for postoperative morbidity and mortality in cirrhotic patients undergoing hepatectomy for HCC [16–19]. Recently, the albumin-bilirubin (ALBI) score was developed to assess the liver function and prognosis of patients with HCC [20, 21]. It’s ability to predict postoperative complications and 30-day mortality following hepatectomy for HCC was also documented [19]. A new scoring system, the RAM (Risk Assessment for early Mortality) score, was also reported to be a powerful beside tool to predict the short-term outcome immediately after hepatectomy for HCC [14]. Despite numerous studies on postoperative outcome, no study to date has investigated the discriminative ability of these scoring systems in predicting early mortality after operation for HCC.

In addition to high mortality rate, high recurrence rate is another important issue that always attract researcher’s attention. Nearly 60% of patients who suffered from HCC relapsed after curative treatment [22]. Due to different etiology, HCC recurrence was generally divided into two types: those occurred within two years after the operation (early recurrence) and those relapsed at least two years after the initial surgery (late recurrence) [23]. It was believed that early recurrence was caused by dissemination of remnant tumor cells after surgical resection and was therefore associated with tumor factors such as large tumor size, presence of vascular invasion, and high α-fetoprotein (AFP) [24]. Late recurrence, on the other hand, was related to underlying chronic liver disease or cirrhosis. In addition to tumor factors, a recent research showed that the ALBI-T score was also a poor prognostic factor for tumor recurrence after liver resection for HCC [25]. Furthermore, the occurrence of postoperative complications was demonstrated to be an independent risk factor for early recurrence after curative hepatectomy for HCC [26]. As a result, tumor, patient, and surgical factors should all be considered for tumor recurrence. Since early recurrence is closed related to the overall survival [27], it would be of vital significance to predict and closely monitor early recurrence postoperatively. Although RAM, ALBI, and MELD scores were all reported to be significantly associated with long-term survival after liver resection [14, 28–35], the effectiveness of these scoring systems to predict early recurrence after liver resection for HCC remained unknown. Therefore, the purpose of the current study was to determine the predictive capability of RAM, ALBI, and MELD scores for the occurrence of early mortality and early recurrence after hepatectomy for HCC.

## Methods

### Patients

We retrospectively reviewed patients with HCC who were treated with curative hepatectomy by our surgical team at Chang Gung Memorial Hospital (CGMH) between 1986 and 2015. After excluding patients who underwent only exploratory laparotomy for liver tumor biopsy, who had distant metastases before operation, who did not have detailed preoperative/intraoperative clinical records, or who did not have regular postoperative out-patient follow-up, a total of 1935 patients were enrolled. The demographics, surgical, and perioperative data were reviewed. The primary endpoints of the study were early (6-month) mortality and early (2-year) recurrence. Tumor staging was obtained based on the American Joint Committee on Cancer (AJCC) TNM staging system for HCC [36]. The preoperative assessment, surgical techniques, postoperative management, and long-term follow-up followed the guidelines published previously [14]. The respective RAM, ALBI, and MELD scores were determined and investigated for their discriminative power in predicting early mortality and recurrence.

### Definition

Major liver resection were defined as resections of three or more liver segments [37]. Major surgical complications comprised of grade III and grade IV surgical complications [38], including postoperative bleeding requiring angiographic embolization or reoperation, major biliary complications requiring drainage or endoscopic intervention, intestinal obstruction requiring operation, upper gastrointestinal bleeding requiring endoscopic hemostasis, massive ascites or pleural effusion requiring paracentesis, sepsis of any etiology, liver failure, renal failure, respiratory failure, or any condition dictating ICU care. Early mortality was defined as the occurrence of death within 6 months after the operation. The cause of early mortality included HCC recurrence/metastasis, hepatic failure due to liver cirrhosis, and postoperative surgical complications. Recurrence was defined as the appearance of characteristic image findings during regular postoperative radiologic examinations and/or elevated serum AFP levels. Early recurrence was defined as the occurrence of recurrence within two years of the initial curative operation [23].

### Computation of scores and classifications

RAM score was obtained by the summation of the scores of 6 independent variables, namely diabetes mellitus (1), albumin ≤3.5 g/dL (2), α-fetoprotein >200 ng/mL (2), major resection (1), blood loss >800 ml (1), and major surgical complications (3). As previously described, RAM classes were developed by visual inspection of the Kaplan-Meier survival curves, and RAM class I, II, and III corresponded to RAM scores of 0–6, 7–9, and 10, respectively [14] (Table 1). Because only 6 cases belonged to RAM class III in the current study, RAM class II and III were combined into RAM class II/III for subsequent analysis. ALBI score was computed by the formula: ALBI = −0.0852 × (albumin g/L) + 0.66 × log (bilirubin μmol/L) [21]. For purposes of analyses, patients were categorized into three groups: grade *I* ≤ −2.60, grade II < −2.60 to ≤ −1.39, and grade III > −1.39 [19, 21]. The standard equation for MELD score was: MELD = 9.57 × ln (creatinine mg/dL) + 3.78 × ln (total bilirubin mg/dL) + 11.2 × ln (INR) + 6.43 [39]. Consistent with previous reports, patients were stratified into three groups based on their MELD scores: grade *I* < 10, grade II 10 to 19, and grade III ≥ 20 [16–18, 35].

**Table 1:** Risk Assessment for early Mortality (RAM)^a^ score for hepatectomy for hepatocellular carcinoma

Variables	Score allocation^b^	Total score	No. (% of total)	6-month mortality (%)	Total score	No. (% of total)	6-month mortality (%)
Diabetes mellitus	1	0	36 (1.8)	1 (2.8)	6	203 (10.5)	27 (13.3)
Albumin ≤ 3.5 g/dL	2	1	36 (1.8)	2 (5.6)	7	112 (5.7)	21 (18.75)
α-fetoprotein > 200 ng/mL	2	2	532 (27.5)	11 (2.1)	8	76 (3.9)	12 (15.8)
Major resection^c^	1	3	308 (15.9)	10 (3.2)	9	39 (2.0)	9 (25.6)
Blood loss > 800 mL	1	4	288 (14.9)	20 (6.9)	10	6 (0.3)	2 (33.3)
Major surgical complications^d^	3	5	299 (15.5)	30 (10)	Total	1935 (100)	145 (7.5)
RAM score^e^	Score			6-month mortality (%)			
Class I	0–6			101 (5.9)			*p* < 0.001
Class II	**7**–9			42 (18.5)			
Class III	10			2 (33.3)			

^a^Risk Assessment for early Mortality score

^b^the regression coefficients (B) were multiplied by two and rounded to integer in order to calculate the RAM score

^c^includes tri-segmentectomy, right/left lobectomy, and extended right/left lobectomy

^d^major surgical complications include grade III-IV surgical complications

^e^AUC = 0.723, *P* < 0.001. When Cutoff score is 4.5, the sensitivity and specificity for 6-month mortality was 0.717 and 0.644, respectively

### Statistical analysis

Fisher’s exact test and Pearson’s χ2 test were used to analyze categorical data. Student’s t test and Mann-Whitney U test were used to analyze continuous variables. The Kaplan-Meier method was employed for survival analysis and the results were compared with the log-rank test. The receiver operating characteristic (ROC) curve was developed to determine the sensitivity and specificity of individual scoring systems. The area under the curve (AUC) value was compared between these systems. Cox regression multivariate analysis was conducted to determine the predictive power of respective scoring system for early mortality and early recurrence. Clinical factors found to be significantly associated with early mortality or recurrence by univariate analysis were included in the multivariate analysis. Results from the multivariate analysis were reported as hazard ratios (HR) and 95% confidence intervals (CI). All calculations were performed with SPSS for windows (SPSS Inc., Chicago, IL, USA). Two-tailed *P*-values less than 0.05 were considered statistically significant.

## Results

### Patient demographics and operative variables

A total of 1935 patients with HCC underwent curative hepatectomy during the study period. The median follow-up time was 41.9 months. Only 189 patients (8.9%) received preoperative treatment, with transarterial chemoembolization (TACE) being the most common preoperative therapy (89.9%). As for operative variables, five hundred and ninety-six (30.8%) patients underwent major liver resection, and 205 (10.6%) patients developed major surgical complications. The detailed clinical and pathological data was summarized in Table 2.

**Table 2:** Demographic data of patients with hepatocellular carcinoma undergoing hepatectomy (*n* = 1935)

Variables^a^	No. (%)	Variables^a^	No. (%)
Gender (Male vs. Female)	1511 (78.1) vs. 424 (21.9)	Surgical complications (major vs. minor/none)^k^	205 (10.6) vs. 1730 (89.4)
HBV^b^ surface antigen (Positive)	1067 (55.2)	Daughter nodule (Yes)	435 (22.5)
Hepatitis C virus (Positive)	568 (29.3)	Cirrhosis (Yes)	984 (50.8)
Non-B Non-C^c^ (Yes)	248 (12.8)	Capsule (Yes)	1552 (80.2)
Child-Pugh Classification (A / B / C)	1863 (96.3) / 27(1.4) / 1(0.1)	Rupture (Yes)	143 (7.4)
Comorbidity (Yes)	739 (38.2)	Vascular invasion (Yes)	693 (35.8)
Diabetes Mellitus (Yes)	405 (20.9)	Variables^a^	Median (IQR)^p^
Hypertension (Yes)	434 (22.4)	Age (year-old)	60 (50–69)
ESRD^d^ (Yes)	38 (2.0)	ICG-15^e^ (%)	7.42 (4.07–12.56)
Smoking (Yes)	453 (23.4)	Albumin (g/dL)	4.19 (3.80–4.40)
Alcohol (Yes)	294 (15.2)	Bilirubin total (mg/dL)	0.7 (0.5–0.9)
Age ≤ 65 (year-old)	1264 (65.3)	Platelet (1000/uL)	172 (129–216)
ICG-15^e^ ≤ 10 (%)	1167 (60.3)	INR^f^	1.1 (1.0–1.1)
Albumin > 3.50 (g/dL)	1678 (86.7)	Creatinine (mg/dL)	1.00 (0.81–1.20)
Bilirubin total ≤ 2.0 (mg/dL)	1894 (97.9)	Pre-OP CEA^l^ (ng/mL)	2.22 (1.35–3.38)
Platelet > 100 (1000/uL)	1688 (87.2)	Pre-OP CA-199^m^ (U/mL)	22.11 (10.63–37.73)
INR^f^ ≤ 1.4	1914 (98.9)	Pre-OP α-fetoprotein^n^ (ng/mL)	23.30 (5.49–328.28)
Pre-OP treatment^g^ (Yes)	189 (9.8)	Tumor size (cm)	3.6 (2.4–6.5)
Pre-OP symptoms^h^ (Yes)	496 (25.6)	OP duration^o^ (minutes)	254 (194–330)
Inflow control^i^ (Yes)	1351 (69.8)	Blood loss (mL)	300 (100–500)
Procedure (Major resection(%))^j^	596 (30.8)		

^a^only patients with available data were analyzed

^b^hepatitis B virus

^c^HCC patients had neither HBV nor HCV infection

^d^end-stage renal disease

^e^indocyanine green retention test at 15 min

^f^international normalized ratio

^g^preoperative treatments included transarterial chemoembolization, percutaneous ethanol injection and radiofrequency ablation

^h^preoperative symptoms included anemia, jaundice, palpable mass and ascites

^i^inflow control included Pringle’s maneuver, Glissonian pedicle control, selective vascular control and total vascular exclusion

^j^major resection included tri-segmentectomy, right/left lobectomy, and extended right/left lobectomy

^k^major surgical complications included grade III-IV surgical complications

^l^preoperative serum carcinoembryonic antigen level

^m^preoperative serum carbohydrate antigen19-9 level

^n^preoperative serum α-fetoprotein level

^o^duration of operation

^p^interquartile range

### Prediction of 6-month (early) mortality

**Fig. 1:**
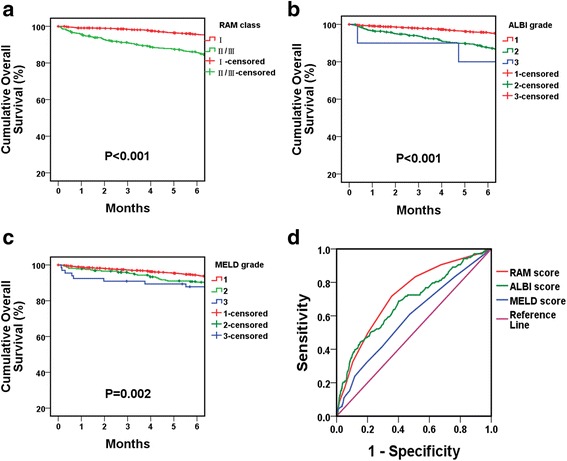
Kaplan–Meier survival curves and ROC curves for early mortality. **a**, **b** and **c** Six-month Kaplan–Meier survival curves according to RAM, ALBI and MELD classifications (all *P* < 0.001). **d** ROC curves of RAM, ALBI and MELD scores for predicting early mortality (AUC =0.723, 0.682, and 0.590, respectively; RAM vs. ALBI, *P* = 0.121, RAM vs. MELD, *P* = <0.001). For RAM score, when cutoff score was 4.5, the sensitivity and specificity for 6-month mortality was 0.717 and 0.644, respectively. For ALBI score, when cutoff score was −2.425, the sensitivity and specificity for 6-month mortality was 0.442 and 0.848, respectively. For MELD score, when cutoff score was 7.5, the sensitivity and specificity for 6-month mortality was 0.609 and 0.528, respectively

By the sixth month after the index operation, a total of 149 patients (7.7%) died due to either postoperative surgical complications, HCC recurrence/metastasis, or complications of liver cirrhosis. The respective RAM, ALBI, and MELD scores were determined for all patients accordingly and summarized in Table 3. The mean RAM, ALBI, and MELD scores for patients who suffered from early mortality vs. those who lived were 5.52 ± 0.17 vs. 3.85 ± 0.05 (*P* < 0.001), −2.47 ± 0.04 vs. -2.81 ± 0.01 (*P* < 0.001), and 9.32 ± 0.33 vs. 8.27 ± 0.07 (*P* = 0.002), respectively. Increasing RAM, ALBI, and MELD scores were associated with an incremental increase in the risk of early mortality. For example, 20.6% (*n* = 48) of RAM class II/III patients died by the sixth month of the index operation while only 5.9% RAM class I patients deceased by the sixth postoperative month. Similar trends were observed among ALBI and MELD grades. To compare the discriminative power of these scoring systems, the ROC curves were formulated and AUC was determined. As shown in Fig. 1, RAM, ALBI, and MELD scores were predictive of early mortality, with AUC of 0.723, 0.682, and 0.590, respectively (all *P* < 0.001). The AUC of RAM was significantly higher than that of MELD (*P* < 0.001), indicating better predictive capability. On the other hand, the AUC of RAM score was comparable to that of ALBI score, with a *P*-value of 0.121. The RAM and ALBI scores should be similarly effective in terms of predicting early mortality as a result.

**Table 3:** Early mortality and early recurrence based on RAM^a^, ALBI^b^ and MELD^c^ score classifications

		Early Mortality vs. Lived	*P*-value	Early recurrence vs. Non-recurrence	*P*-value
RAM score^a^ (mean ± SE)		5.52 ± 0.17 vs. 3.85 ± 0.05	<0.001	4.34 ± 0.06 vs.3.35 ± 0.06	<0.001
ALBI score^b^ (mean ± SE)		−2.47 ± 0.04 vs. -2.81 ± 0.01	<0.001	−2.71 ± 0.01 vs. -2.86 ± 0.01	<0.001
MELD score^c^ (mean ± SE)		9.32 ± 0.33 vs. 8.27 ± 0.07	0.002	8.54 ± 0.09 vs. 8.23 ± 0.10	0.113
	Number (% of total)	Early mortality (6-month) (%)	*P*-value	Early recurrence (2-year) (%)	*P*-value
RAM score^a^ (n = 1935)			<0.001		<0.001
RAM class I	1702 (88)	101 (5.9)		887 (52.6%)	
RAM class II/III	233 (12)	48 (20.6)		148 (68.2%)	
ALBI score^b^ (*n* = 1892)			<0.001		<0.001
ALBI grade 1	1341 (70.9)	67 (5.0)		666 (49.7%)	
ALBI grade 2	541 (28.6)	75 (13.9)		339 (62.7%)	
ALBI grade 3	10 (0.5)	2 (20.0)		7 (70.0%)	
MELD score^c^ (*n* = 1898)			0.002		0.106
MELD grade 1	1513 (79.7)	97 (6.4)		799 (53.5%)	
MELD grade 2	319 (16.8)	37 (11.6)		183 (58.7%)	
MELD grade 3	66 (3.5)	8 (12.1)		28 (45.9%)	

^a^Risk Assessment for early Mortality score

^b^albumin–bilirubin score

^c^Model for End-Stage Liver Disease score

After Cox regression multivariate analysis, only RAM and ALBI grades remained independently associated with an increased risk of 6-month mortality (Table 4). MELD grade, on the other hand, failed to independently predict the occurrence of early mortality. As shown in Table 4, RAM class II/III was significantly associated with an increased risk of early mortality (HR 2.847, 95% CI 1.884~4.302, *P* < 0.001). While ALBI grade II had a significantly increased risk of early mortality (HR 2.309, 95% CI 1.577~3.383, *P* < 0.001), ALBI grade III failed to independently predict early mortality (*P* = 0.953). RAM score was demonstrated to be a more preferable tool in predicting early mortality.

**Table 4:** Cox regression multivariate analyses of factors associated with early mortality and early recurrence in hepatocellular carcinoma after hepatectomy

	Multivariate analysis	
	Hazard ratio (95% CI)^j^	*P* -value
Early Mortality		
RAM score^a^		
class II/III vs. class I	2.847 (1.884~4.302)	<0.001
ALBI score^b^		<0.001
grade 2 vs. grade 1	2.309 (1.577~3.383)	<0.001
grade 3 vs. grade 1	N/A^k^	0.953
MELD score^c^		0.271
grade 2 vs. grade 1	1.218 (0.782~1.897)	0.384
grade 3 vs. grade 1	1.873 (0.830~4.231)	0.131
Alcohol consumption (Yes)	1.799 (1.105~2.930)	0.018
Cigarette smoking (Yes)	1.110 (0.710~1.734)	0.648
Age > 80 (year-old)	1.988 (0.907~4.354)	0.086
Pre-OP symptoms^d^ (Yes)	1.411 (0.956~2.083)	0.083
Pre-OP treatment^e^ (Yes)	1.271 (0.765~2.111)	0.355
Pre-OP platelets < 100 (1000/uL)	1.453 (0.905~2.332)	0.122
Pre-OP total bilirubin > 2.0 (mg/dL)	1.377 (0.469~4.039)	0.561
Pre-OP hemoglobin < 10 (g/dL)	1.102 (0.596~2.038)	0.756
ICG-15^f^ > 10 (%)	1.081 (0.740~1.580)	0.687
OP duration^g^ > 270 (mins)	1.295 (0.875~1.915)	0.196
Tumor size > 10 (cm)	1.770 (1.130~2.771)	0.013
Early Recurrence		
RAM score^a^		
class II/III vs. class I	1.640 (1.352~1.988)	<0.001
ALBI score^b^		0.001
grade 2 vs. grade 1	1.382 (1.190~1.606)	<0.001
grade 3 vs. grade 1	0.962 (0.301~3.077)	0.948
MELD score^c^		0.868
grade 2 vs. grade 1	0.952 (0.787~1.150)	0.608
grade 3 vs. grade 1	1.015 (0.683~1.509)	0.941
Alcohol consumption (Yes)	1.249 (1.003~1.557)	0.047
Cigarette smoking (Yes)	1.258 (1.057~1.498)	0.010
Pre-OP symptoms^d^ (Yes)	1.006 (0.855~1.184)	0.943
Pre-OP treatment^e^ (Yes)	1.183 (0.953~1.469)	0.127
Pre-OP total bilirubin > 2.0 (mg/dL)	1.513 (0.921~2.487)	0.102
Pre-OP hemoglobin < 10 (g/dL)	1.215 (0.903~1.634)	0.199
Pre-OP ALT^h^ > 40 (U/L)	1.077 (0.940~1.234)	0.284
ICG-15^f^ > 10%	1.347 (1.167~1.554)	<0.001
Tumor size > 10 (cm)	1.773 (1.456~2.160)	<0.001
Inflow control^i^ (Yes)	1.162 (0.985~1.370)	0.075

^a^Risk Assessment for early Mortality score

^b^albumin–bilirubin score

^c^Model for End-Stage Liver Disease score

^d^preoperative symptoms included anemia, jaundice, palpable mass and ascites

^e^preoperative treatments included transarterial chemoembolization, percutaneous ethanol injection and radiofrequency ablation

^f^indocyanine green retention test at 15 min

^g^duration of operation

^h^preoperative serum alanine aminotransferase level

^i^inflow control included as Pringle’s maneuver, Glissonian pedicle control, selective vascular control and total vascular exclusion

^j^95% confidence interval

^k^Not Applicable

### Prediction of 2-year (early) recurrence

**Fig. 2:**
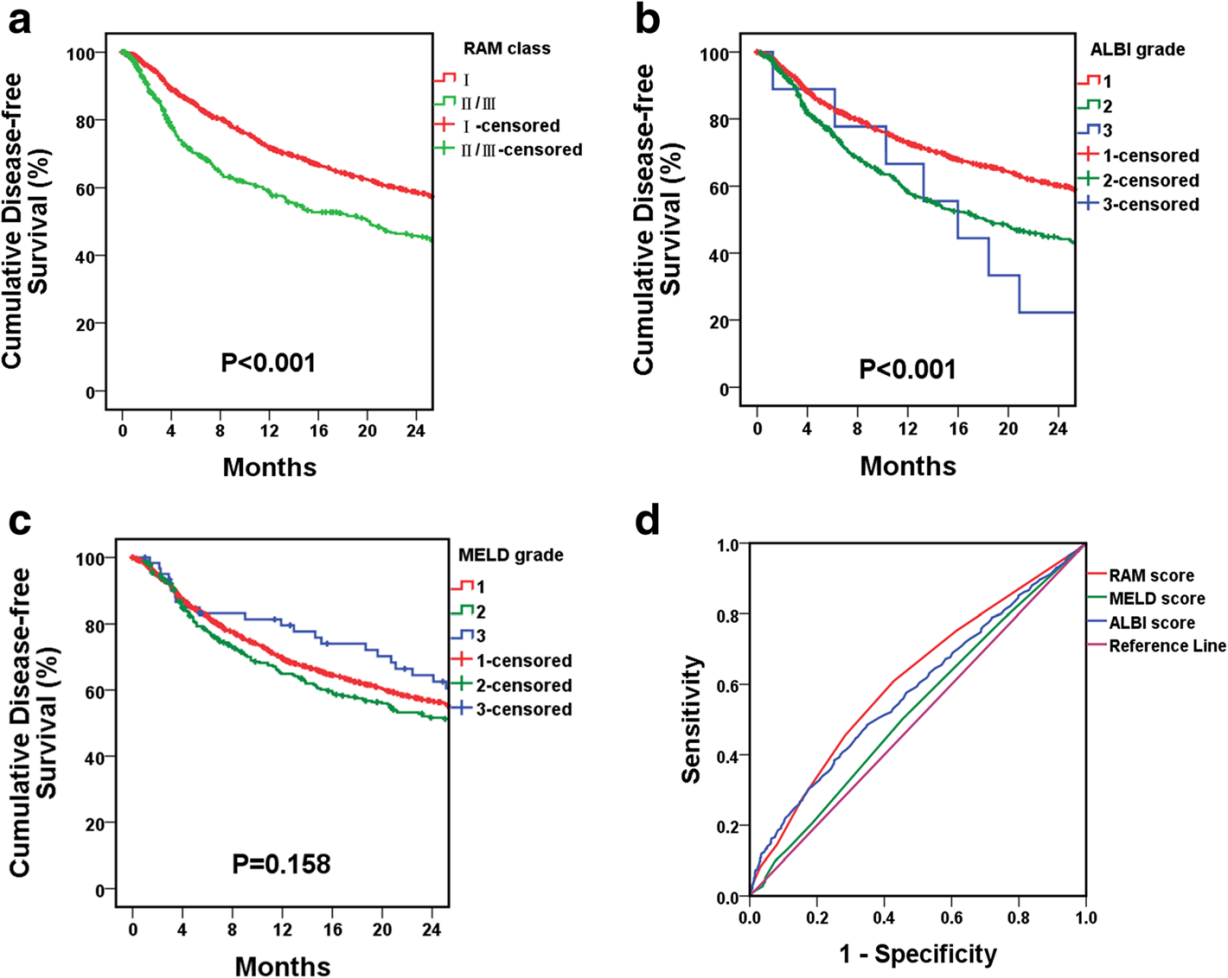
Kaplan–Meier disease-free survival curves and ROC curves for early recurrence. **a**, **b** and **c** Two-year Kaplan–Meier disease free survival curves according to RAM, ALBI and MELD classifications (*P* < 0.001, *P* < 0.001 and *P* = 0.158, respectively). **d** ROC curves of RAM, ALBI and MELD scores for predicting early recurrence (AUC =0.611, 0.582, and 0.527, respectively; RAM vs. ALBI, *P* = 0.057, RAM vs. MELD, *P* = 0.002). For RAM score, when cutoff score was 3.5, the sensitivity and specificity for early recurrence was 0.609 and 0.573, respectively. For ALBI score, when cutoff score was −2.755, the sensitivity and specificity for early recurrence was 0.485 and 0.651, respectively. For MELD score, when cutoff score was 7.5, the sensitivity and specificity for early recurrence was 0.502 and 0.545, respectively

A thousand and thirty-five patients (53.5%) developed recurrence within two years of the index operation. The respective RAM, ALBI, and MELD scores were determined and summarized in Table 3. The mean RAM, ALBI, and MELD scores for patients who developed early recurrence vs. those who did not were 4.34 ± 0.06 vs.3.35 ± 0.06 (*P* < 0.001), −2.71 ± 0.01 vs. -2.86 ± 0.01 (*P* < 0.001), and 8.54 ± 0.09 vs. 8.23 ± 0.10 (*P* = 0.113), respectively. Increasing RAM and ALBI scores were associated with an increase in early recurrence. For example, 68.2% (*n* = 148) of RAM class II/III patients developed recurrence by the 2nd year of the index operation while 52% RAM class I patients had recurrence by the 2nd year. ALBI grade also had similar association with the development of early recurrence. In contrast, MELD grade was not associated with the occurrence of early recurrence after curative hepatectomy. Figure 2 demonstrated the DFS curves and ROC curves of respective scoring systems. The AUC of RAM, ALBI, and MELD scores for early recurrence were 0.611, 0.582, and 0.527, respectively (*P* < 0.001 for RAM and ALBI, and 0.044 for MELD). The AUC of RAM was much higher than that of both ALBI and MELD (RAM vs. ALBI, *P* = 0.057; RAM vs. MELD *P* = 0.002), indicating that RAM score may be a better scoring system in terms of predicting early recurrence.

On Cox regression multivariate analysis, after adjusting for other significant clinical risk factors for early recurrence, RAM class, ALBI grade, cigarette smoking, alcohol consumption, tumor size >10 cm, and ICG-15 > 10% remained independently associated with an increased risk of early recurrence (*P* < 0.001, 0.001, 0.010, 0.047, <0.001, and <0.001, respectively) (Table 4). RAM class II/III had a 1.6-fold risk for developing early recurrence (95% CI 1.352 ~ 1.988, *P* < 0.001), and ALBI grade II was 1.4 times more likely to have early recurrent HCC after liver resection (95% CI 1.190 ~ 1.606, *P* < 0.001). Due to higher AUC, RAM score should be a superior tool in predicting early recurrence.

## Discussion

The current study demonstrated that the RAM scoring system was significantly better than MELD score in terms of predicting early mortality and early recurrence after liver resection for HCC. Although RAM and ALBI scores seemed to be comparable to each other, we believe the RAM scoring system is superior to ALBI score for several reasons. First, the RAM score had a higher AUC when predicting both early mortality and early recurrence. Second, while ALBI grade III failed to predict early mortality, RAM class II/III was proved to be independently associated with early mortality. Third, unlike ascites and encephalopathy included in Child-Pugh classification, the variables incorporated in RAM scoring system were all objectively determined and thus introduced less bias [21, 28, 30]. Lastly, the RAM scoring system incorporated patient, surgical, and tumor factors into considerations, rendering RAM score superior to other major scoring systems.

For patient factors, diabetes mellitus is a well-known risk factor for major surgery because of the susceptibility to infection [40]. A previous study demonstrated that diabetes mellitus impaired hepatic regeneration and decreased hepatic intracellular energy status after partial hepatectomy in rats [41]. Non-diabetic status and ICG-15 < 20% has been considered a safe limit for bisegmentectomy [40, 42]. Albumin level, on the other hand, reflects general nutritional status and liver function and has been employed in most scoring systems such as Child-Pugh classification, RAM, and ALBI scores. These patient factors are believed to be of paramount significance for liver surgery and may influence the postoperative outcome.

In addition to patient factors, surgical factors should also play important roles in determining postoperative outcome. Previous studies have shown that massive blood loss and blood transfusions were associated with adverse effects on the immune system, leading to an increased risk of postoperative infection, complications, and mortality [3, 12, 14, 40, 42–44]. Minimization of intraoperative blood loss, on the other hand, was an widely accepted manner to prevent postoperative complications [42]. Furthermore, our recent study found that major liver resection and major surgical complications were two independent risk factors for 6-month mortality, with major surgical complications being the most significant one [14]. These and other evidence indicated that surgical factors should definitely affect postoperative outcome including the occurrence of early mortality. RAM score, as a result, should be a preferable scoring system in predicting early mortality.

As for postoperative tumor recurrence, previous research reported that risk factors for early recurrence included large tumor size, multiple tumors, vascular invasion, poor tumor differentiation, and high AFP level [26, 27, 45, 46]. In addition to tumor factors, we believe that surgical factors such as surgical complications should also play significant roles in promoting tumor recurrence. Nevertheless, few studies to date had discussed the influence of surgical factors on early HCC recurrence after operation. One of the studies that investigated the impact of surgery reported that postoperative complication was a predictive factor for early HCC recurrence [26]. One possible explanation for this result should be immunosuppression, which allows residual tumor cells or micrometastasis from the primary tumor to further proliferate and survive in the host [27, 45–47]. In addition to HCC, there were other cancers whose oncological outcome and survival were demonstrated to be significantly influenced by surgical complications. These cancer included lung cancer, rectal cancer, esophageal cancer, gastric cancer, pancreatic cancer, and hilar cholangiocarcinoma [26, 48–50]. Therefore, we believe that surgical factors should be as important as tumor factors in promoting early HCC recurrence and should be incorporated into scoring system. RAM score, subsequently, is a more comprehensive evaluation system and should be superior to ALBI and MELD scores in terms of predicting early mortality and early recurrence.

Despite significant results, this study still had several limitations. First, since it is a retrospective analysis based on clinical data retrieved from the database, incomplete data were inevitable when reviewing records from earlier days. Second, the relatively long enrollment period might introduce bias since surgical techniques and perioperative care may have improved during this period of time. Third, very few patients were categorized as either RAM class III, ALBI grade III, or MELD grade III, resulting in their poor predictive capability. Further adjustments are thus warranted to improve the predictive efficacy of these scoring systems. Fourth, since HBV viral load has been shown to be associated with shorter DFS and OS after hepatectomy [51], and pathological factors such as vascular invasion and daughter nodules were well-known risk factors for early HCC recurrence, a modified RAM score incorporating these factors should be sought to enhance the predictive power for early recurrence.

## Conclusions

Our study demonstrated that RAM score is an effective and user-friendly bedside scoring system to predict early mortality and early recurrence after hepatectomy for HCC. In addition, the discriminative capability of RAM score is superior to ALBI and MELD scores since it incorporates patient, tumor, and surgical factors into consideration. Further study is warranted to investigate the mechanism by which surgical factors may influence postoperative oncological outcome and to validate our findings.

## Abbreviations

AFP: α-fetoprotein
AJCC: American Joint Committee on Cancer
ALBI score: Albumin-bilirubin score
AUC: area under the ROC curve
CGMH: Chang Gung Memorial Hospital
CKD: Chronic kidney disease
C-P score: Child–Pugh score
CT: Computed tomography
DM: Diabetes mellitus
HBV: Hepatitis B virus
HCC: Hepatocellular carcinoma
HCV: Hepatitis C virus
ICG-R15: Indocyanine green retention test at 15 min
ICU: Intensive care unit
MELD score: The model for end-stage liver disease score
MRI: Magnetic resonance imaging
RAM score: Risk assessment for early mortality score
ROC curve: Receiver operating characteristic curve
TIPS: Transjugular intrahepatic portosystemic shunts
